# A Hybrid Mesenchymal-Stem-Cell-Derived Decellularized Matrix Scaffold Supports Bone Repair and Vascular Perfusion in Steroid-Associated Osteonecrosis

**DOI:** 10.34133/bmr.0383

**Published:** 2026-06-29

**Authors:** Yijun He, Chu Hua, Lin Liang, Chen Huang, Yuan Li, Jiongfeng Huang, Cheng Luo, Zhi-Yong Zhang

**Affiliations:** ^1^ Department of Osteoarthropathy and Sports Medicine, The Affiliated Panyu Central Hospital, Guangzhou Medical University, Guangzhou, Guangdong 511400, PR China.; ^2^ Translational Research Centre of Regenerative Medicine and 3D Printing, Department of Orthopaedic Surgery, Guangzhou Key Laboratory of Spine Disease Prevention and Treatment, Guangdong Province Engineering Research Center for Biomedical Engineering, State Key Laboratory of Respiratory Disease, Guangdong Provincial Key Laboratory of Major Obstetric Diseases, Guangdong Provincial Clinical Research Center for Obstetrics and Gynecology, The Third Affiliated Hospital, Guangzhou Medical University, Guangzhou, Guangdong 510150, PR China.; ^3^ Department of Minimally Invasive Interventional Radiology, The Affiliated Panyu Central Hospital, Guangzhou Medical University, Guangzhou, Guangdong 511400, PR China.

## Abstract

Steroid-associated osteonecrosis (SAON) is characterized by glucocorticoid-associated vascular compromise, impaired bone repair, and a dysregulated inflammatory microenvironment. Although core decompression (CD) remains the main joint-preserving procedure, its efficacy is often limited by the hostile local niche. Here, we engineered a hybrid CDM@Fibrin/poly(ε-caprolactone) (PCL) scaffold by incorporating human umbilical cord mesenchymal-stem-cell-derived decellularized matrix (CDM) into a 3D-printed PCL framework. Proteomic profiling showed enrichment of extracellular-matrix-associated proteins linked to focal adhesion, extracellular matrix–receptor interaction, and phosphatidylinositol 3-kinase–Akt-related signaling. In vitro, solubilized CDM was biocompatible and modulated macrophage behavior in a context-dependent manner; under basal conditions, its effects on canonical polarization markers were modest, whereas under inflammatory challenge it attenuated lipopolysaccharide-induced M1-like activation and partially restored pro-healing features. In a rat femoral condyle defect model, CDM@Fibrin/PCL enhanced bone formation and was associated with lower CD86 and relatively higher CD206 signals than Fibrin/PCL controls. In a preclinical SAON model, scaffold-augmented CD markedly improved new bone formation and perfused vascular volume relative to CD alone. Exploratory transcriptomic analysis identified pathway-level associations related to immune regulation, extracellular matrix remodeling, and reparative signaling. Collectively, these findings suggest that mesenchymal-stem-cell-derived CDM functions as a bioactive matrix component that helps rebalance the local inflammatory niche and supports bone repair with improved vascularization-related outcomes in SAON.

## Introduction

Steroid-associated osteonecrosis (SAON) represents a debilitating, nontraumatic bone pathology typically triggered by the administration of high-dose or prolonged glucocorticoid regimens [1]. This condition predominantly affects young and middle-aged populations, frequently culminating in the collapse of the femoral head and subsequent lifelong physical impairment. In the USA alone, the annual incidence is estimated at 10,000 to 20,000 new cases [2], while in China, the patient population suffering from femoral head necrosis has surpassed 8 million, with SAON contributing to roughly 25% of these [3]. More recently, the COVID-19 pandemic has intensified this clinical challenge; the widespread clinical reliance on corticosteroids to manage severe infections is expected to drive a global surge in SAON cases [4]. Such statistics underscore a pressing, unmet clinical demand for innovative, joint-preserving interventions capable of addressing the complex pathophysiology inherent to SAON.

The administration of glucocorticoids sets off a multifaceted cascade of musculoskeletal insults [5]. These agents provoke adipocyte hypertrophy within the bone marrow, promote lipid deposition, and induce microvascular thrombosis—factors that collectively impair perfusion to the femoral head. Furthermore, excessive steroid exposure triggers osteocyte apoptosis and suppresses osteoblastogenesis [6,7]. Clinical data suggest that long-term treatment leads to osteonecrosis in approximately 9% to 40% of patients, with the programmed death of osteocytes serving as a hallmark histopathologic feature. This loss of viable osteocytes diminishes vascular endothelial growth factor expression and impairs angiogenesis, thereby compromising the mechanical integrity of bone and precipitating subchondral collapse. Although glucocorticoids are widely used as anti-inflammatory agents, prolonged exposure can paradoxically contribute to a chronic dysregulated marrow microenvironment associated with inflammation, vascular compromise, and impaired bone repair [6,8–13]. Macrophages are central regulators of this process. Early-stage lesions are often characterized by infiltration of pro-inflammatory macrophages and elevated levels of cytokines such as tumor necrosis factor-alpha (TNF-α), interleukin-1 beta (IL-1β), and interleukin-6 (IL-6), which promote osteoclastogenesis and further disrupt bone regeneration [14]. By contrast, macrophages with tissue-repair-associated phenotypes can help suppress inflammation and support angiogenesis and osteogenesis [15]. Thus, successful regeneration in SAON likely requires not only mechanical support and revascularization but also rebalancing of the local inflammatory niche.

Core decompression (CD) remains the primary surgical strategy for early-stage SAON. By creating channels within the necrotic femoral head, CD lowers intramedullary pressure and encourages revascularization, providing symptomatic relief. However, its long-term efficacy is inconsistent, in part because decompression does not sufficiently reverse the hostile biochemical and immunological environment of the osteonecrotic lesion. Even when supplemented with bone grafts, some patients still experience femoral head collapse within several years [16]. While adjuvant therapies like bisphosphonates, statins, or anticoagulants have been utilized, they offer only marginal bone preservation. Various osteoconductive materials—including porous tantalum rods, calcium-phosphate cements, and 3D-printed scaffolds—have been employed to provide mechanical reinforcement [17]. Although these implants can improve local blood flow and structural stability, their clinical efficacy remains hampered by a lack of active immunomodulatory capacity [18]. This limitation is especially important in SAON, where chronic inflammation and vascular insufficiency are key barriers to repair.

Recent breakthroughs in “immuno-instructive” biomaterials have shifted the focus toward scaffolds that actively modulate the host immune response to promote bone repair [12,19–21]. For example, scaffolds engineered for the sustained release of interleukin-4 (IL-4) have demonstrated the ability to polarize macrophages toward an M2 phenotype, thereby accelerating healing in bone defect models [22]. Other strategies involve decorating implant surfaces with signaling molecules like CD47 to minimize phagocytosis and enhance biocompatibility [23]. These developments reflect a growing consensus that effective bone regeneration requires more than a physical frame; it necessitates a pro-healing immune microenvironment [21]. For SAON, this concept is especially attractive because the pathological niche is defined by both inflammation and impaired vascular regeneration.

Mesenchymal stem cells (MSCs) are renowned for their potent immunomodulatory effects, much of which is mediated through their secreted extracellular matrix (ECM) [24]. The ECM generated during MSC culture—often termed cell-derived decellularized extracellular matrix (CDM)—comprises a sophisticated network of collagen, fibronectin, laminin, and glycosaminoglycans [25]. Far from being a mere structural scaffold, this matrix functions as a bioactive reservoir for growth factors and cytokines that direct cellular behavior [26]. Notably, the decellularized MSC-matrix retains essential proteins even after the cellular components are removed [27,28]. Our previous work demonstrated that MSC-laden microtissues enriched in native matrix promoted tissue repair and were associated with favorable macrophage responses [29]. Furthermore, independent studies have confirmed that MSC-CDM exerts immunomodulatory influences that bolster musculoskeletal regeneration [30–33]. These observations suggest that CDM may serve as a promising cell-free bioactive component for engineering regenerative microenvironments.

Despite this potential, several important gaps remain. First, few studies have investigated whether MSC-derived CDM can be integrated into a mechanically competent scaffold suitable for load-bearing orthopedic applications. Second, although CDM has been linked to immunomodulatory activity, its role in the specific inflammatory setting of SAON remains insufficiently defined. Third, no previous work has systematically examined the combination of CDM-based scaffold implantation with CD in a preclinical SAON model while simultaneously evaluating bone regeneration, vascularization, and transcriptomic changes in the defect microenvironment.

**Fig. 1.**
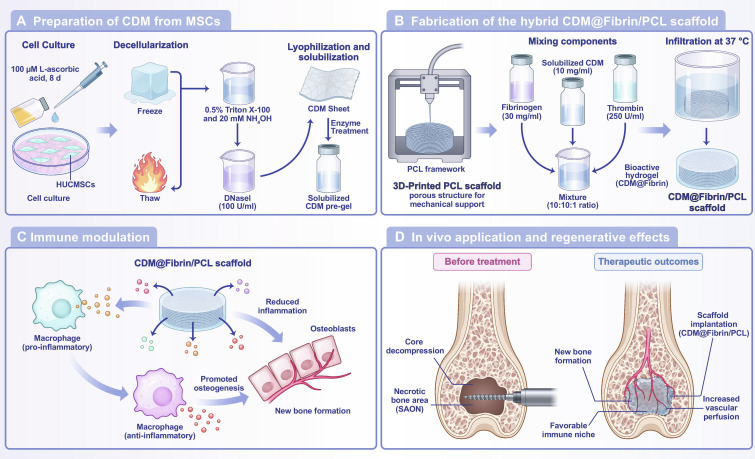
Schematic illustration of the preparation, fabrication, immunomodulatory function, and in vivo application of the CDM@Fibrin/poly(ε-caprolactone) (PCL) scaffold for steroid-associated osteonecrosis (SAON). (A) Preparation of cell-derived decellularized extracellular matrix (CDM) from mesenchymal stem cells (MSCs): Cells are cultured to confluence with ascorbic acid supplementation to enhance extracellular matrix (ECM) deposition, followed by decellularization via freeze–thaw cycles, incubation with 0.5% Triton X-100 and 20 mM NH_4_OH, and DNase I treatment to remove cellular remnants, resulting in a lyophilized CDM suitable for solubilization into a pre-gel solution. HUCMSC, human umbilical cord mesenchymal stem cell. (B) Fabrication of the hybrid CDM@Fibrin/PCL scaffold: A porous PCL framework is 3D printed for mechanical support, then infiltrated with a mixture of fibrinogen (30 mg/ml), solubilized CDM (10 mg/ml), and thrombin (250 U/ml) in a 10:10:1 ratio to form a bioactive hydrogel that fills the scaffold pores, ensuring structural integrity and bioactivity. (C) CDM modulates macrophage activation, attenuates pro-inflammatory responses, and supports a more pro-healing microenvironment. (D) In vivo application in a bone defect model demonstrating immunoregulation and vascularized bone regeneration.

To address these gaps, we developed a hybrid CDM@Fibrin/poly(ε-caprolactone) (PCL) scaffold by incorporating human umbilical cord MSC-derived CDM into a 3D-printed PCL framework filled with fibrin hydrogel [34,35]. We hypothesized that the inclusion of CDM would help modulate the inflammatory microenvironment, particularly under pro-inflammatory conditions, and thereby support angiogenesis and bone regeneration after CD. To test this hypothesis, we combined proteomic characterization, in vitro biocompatibility and macrophage assays, in vivo bone defect and SAON models, and transcriptomic profiling of the regenerative niche. Through this integrated approach, we aimed to evaluate whether a CDM-based immuno-instructive scaffold could improve the biological performance of joint-preserving treatment in SAON (Fig. 1).

## Materials and Methods

### Preparation and characterization of cell-derived matrix

#### Cell-derived-matrix isolation and decellularization

Primary human umbilical cord MSCs were purchased from Cyagen (China). The cells were cultured at 37 °C in a humidified atmosphere containing 5% CO_2_, using Dulbecco's Modified Eagle's Medium/Ham's F-12 medium supplemented with 10% fetal bovine serum, 100 U/ml penicillin, 100 μg/ml streptomycin, and 1% Glutamax (Gibco, USA). The medium was changed every 2 d. Cells at passages 5 to 6 were used for the preparation of cell-derived matrix (CDM), as previously described. Cells were cultured until they reached approximately 90% confluence, at which point 100 μM L-ascorbic acid was added to the medium, and the cells were cultured for an additional 8 d.

To decellularize the CDM, we adhered to established protocols [28] by subjecting the cell layer to 3 consecutive freeze–thaw cycles, followed by incubation with an extraction buffer containing 0.5% Triton X-100 and 20 mM NH_4_OH (Sigma-Aldrich) in phosphate-buffered saline (PBS, pH 7.4) at 37 °C for 5 min. The samples were then rinsed with PBS 5 times until the pH of the final rinse was neutral. Next, the samples were treated with DNase I (100 U/ml in PBS containing calcium and magnesium ions; Beyotime) for 1 h at 37 °C. After decellularization, the CDM was washed 5 times with deionized water to remove residual DNase. The matrix was then scraped from the culture surface and lyophilized before use. As a control, cell sheets scraped off without decellularization were collected for subsequent processing.

#### Histological and immunofluorescence

To characterize the major components of the matrix, samples collected before and after decellularization were fixed in 4% paraformaldehyde for 30 min, embedded in paraffin, and sectioned. Slides were stained with hematoxylin and eosin (H&E) and Masson’s trichrome. Immunofluorescence staining was also performed: Sections were incubated overnight at 4 °C with primary antibodies against collagen I (1:200; Abcam, Ab6308) and vimentin (1:100; Abcam, Ab92547), followed by incubation with secondary antibodies for 1 h and counterstaining with 4',6-diamidino-2-phenylindole (DAPI) for 5 min. Images were acquired using a fluorescence microscope (Olympus Imaging Systems, Japan).

#### Scanning electron microscopy

Cell sheets before and after decellularization were lyophilized and then coated with gold before scanning electron microscopy (SEM) analysis (Hitachi, Japan). The operating voltage was set to 10.0 kV.

#### SDS–PAGE protein profiling

To analyze the protein composition of the CDM, 10 μg of protein extract mixed with 4× loading buffer was loaded onto a 1.5-mm, 4% to 12% gradient sodium dodecyl sulfate–polyacrylamide gel electrophoresis (SDS–PAGE) gel. Electrophoresis was performed at 120 V for at least 100 min. The gel was stained with Coomassie Blue overnight and destained in deionized water containing 10% acetic acid and 40% ethanol for 2 h. Protein bands were visualized using a GE Image Scanner III densitometer, and images were captured with ImageQuant TL software (GE Healthcare).

#### Proteomics and bioinformatic analysis

For peptide identification, liquid chromatography/mass spectrometry analysis was performed on CDM samples (*n* = 3). Samples were homogenized in 4% SDS and 100 mM dithiothreitol and lysed using a filter-aided sample preparation protocol. Proteins were classified according to Gene Ontology (GO) annotations in the categories of biological processes, cellular components, and molecular functions. Kyoto Encyclopedia of Genes and Genomes (KEGG) pathway enrichment analysis was performed to identify signaling pathways associated with the identified proteins.

#### Quantification of residual DNA

To quantify the removal of nucleic acids, DNA was extracted using a Genomic DNA Kit (Solarbio, China), and concentrations were measured by absorbance at 260 nm using a Nanodrop spectrophotometer (Thermo Fisher Scientific, USA).

### In vitro biocompatibility and functional assays

#### Cell culture and viability/cytotoxicity evaluation assays

Human bone-marrow-derived MSCs (UE7T-13) and mouse macrophages (RAW264.7) were purchased from Meishen CTCC. Cells were cultured at 37 °C in a humidified atmosphere with 5% CO_2_, using high-glucose DMEM supplemented with 10% fetal bovine serum, 100 U/ml penicillin, and 100 μg/ml streptomycin. The optimal concentration of CDM was determined using a Cell Counting Kit-8 (CCK-8; Dojindo Laboratories, Japan) assay. UE7T-13 cells were seeded at a density of 3 × 10^3^ cells per well, and RAW264.7 cells at 8 × 10^3^ cells per well, in 96-well plates. CDM was added to the culture medium at final concentrations of 0, 10, 50, and 100 μg/ml. After incubation with CCK-8 for 2 h at 37 °C, absorbance was measured at 450 nm. Based on these results, a CDM concentration of 50 μg/ml was used for subsequent assays.

#### Macrophage polarization assays

RAW264.7 cells were allocated into 5 experimental groups: (a) untreated control; (b) IL-4 (M2 positive control; 20 ng/ml for 24 h); (c) lipopolysaccharide (LPS) (M1 positive control; 100 ng/ml for 24 h); (d) CDM alone (50 μg/ml for 24 h); and (e) LPS-CDM, in which cells were preconditioned with LPS as above, followed by CDM (50 μg/ml for 24 h). For flow cytometric analysis, cells were gently detached using enzyme-free dissociation buffer and blocked with Fc block (Biolegend, USA, Cat#422301). Surface markers were stained using fluorophore-conjugated anti-CD86 and anti-CD206 antibodies (Biolegend, USA, Cat#105007 and Cat#141703), and a live/dead viability dye (Biolegend, USA, Cat#423117) was added to exclude nonviable cells. Data acquisition was performed using the flow cytometer Attune NxT (Thermo Fisher Scientific), and results were analyzed with FlowJo software. The gating strategy included sequential selection of forward scatter/side scatter singlets, live cells, and subsequent identification of CD86^+^ and/or CD206^+^ subsets. All experiments were conducted in 3 independent biological replicates with technical duplicates.

#### Real-time quantitative polymerase chain reaction

Total RNA was isolated from cells using Trizol reagent (Thermo Fisher Scientific) according to the manufacturer’s protocol. RNA purity was assessed by measuring the OD_260_/OD_280_ ratio (>1.8) using a BioPhotometer plus (Eppendorf, Germany), and integrity was verified by 1.5% agarose gel electrophoresis. Complementary DNA (cDNA) was synthesized using EasyScript First-Strand cDNA Synthesis SuperMix (TransGen Biotech, China) with the following thermocycling conditions: 25 °C for 10 min, 42 °C for 30 min, and 85 °C for 5 s. Quantitative polymerase chain reaction (qPCR) was performed using 2× SYBR Green qPCR SuperMix (TransGen Biotech) on an ABI PRISM 7500 Real-Time PCR System (Thermo Fisher Scientific). The target genes analyzed included iNOS (Nos2), IL-6 (Il6), TNF-α (Tnf), IL-1β (Il1b), Arg1, IL-10 (Il10), CD206 (Mrc1), and CD163, with β-actin as the housekeeping gene. Primer sequences are listed in Table S1. The qPCR cycling conditions were as follows: 95 °C for 5 min, followed by 40 cycles of 95 °C for 15 s and 60 °C for 32 s with fluorescence acquisition. Melt curve analysis was performed from 60 to 95 °C. Relative gene expression levels were calculated using the 2^−ΔΔCt^ method, and all reactions were conducted in triplicate.

#### Immunofluorescence

RAW264.7 cells were seeded on sterile glass coverslips and cultured under the indicated treatment conditions: untreated control, IL-4 (20 ng/ml, 24 h), LPS (100 ng/ml, 24 h), CDM (50 μg/ml, 24 h), and LPS-CDM, in which cells were first stimulated with LPS (100 ng/ml, 24 h) and then treated with CDM (50 μg/ml, 24 h). After treatment, cells were fixed with 4% paraformaldehyde for 15 min at room temperature, permeabilized with 0.1% Triton X-100 for 10 min, and blocked with 5% bovine serum albumin for 1 h. Samples were then incubated overnight at 4 °C with primary antibodies against CD86 (1:100; TENNG, TNG36-12615) and CD206 (1:400; TENNG, TNG26-355250), followed by incubation with species-appropriate fluorophore-conjugated secondary antibodies for 1 h at room temperature in the dark. Nuclei were counterstained with DAPI. Fluorescence images were acquired using a confocal microscope under identical laser power, gain, and exposure settings for all groups. For quantitative analysis, at least 3 random fields per coverslip were imaged from 3 independent experiments. Mean fluorescence intensity of CD86 and CD206 was measured using ImageJ software by blinded assessors after background subtraction. The fluorescence signal of each marker was normalized to the corresponding DAPI-positive area/cell number, and the ratios of CD86/DAPI, CD206/DAPI, and CD206/CD86 were calculated for comparison among groups.

#### Western blot analysis

RAW264.7 cells were treated as described above: control, IL-4 (20 ng/ml, 24 h), LPS (100 ng/ml, 24 h), CDM (50 μg/ml, 24 h), and LPS-CDM. Total protein was extracted using RIPA buffer containing protease inhibitors (Beyotime, China) on ice for 30 min, followed by centrifugation at 12,000×*g* for 15 min at 4 °C. The supernatants were collected, and protein concentration was measured using a BCA assay kit (Beyotime, China). Equal amounts of protein (20 to 30 μg) were separated by 10% SDS–PAGE and transferred to PVDF membranes (Millipore, USA). Membranes were blocked with 5% nonfat milk in Tris-buffered saline–Tween 20 for 1 h at room temperature and then incubated overnight at 4 °C with primary antibodies against CD86 (fn-test; #FNab01504, 1:1,000), CD206 (CST; #24595S, 1:1,000), and glyceraldehyde 3-phosphate dehydrogenase (GAPDH; Shanghai Kangcheng, #KC-5G5, 1:10,000). After washing with Tris-buffered saline–Tween 20, membranes were incubated with horseradish-peroxidase-conjugated secondary antibodies, including Goat Anti-Rabbit IgG(H+L) (Southern Biotech, #4050-05, 1:20,000) for CD206 and CD86 detection and Rabbit Anti-Mouse IgG(H+L)–horseradish peroxidase (Southern Biotech, #6170-05, 1:10,000) for GAPDH detection, for 1 h at room temperature. Protein bands were detected using ECL reagent (Millipore, #WBKLS0500) and imaged using x-ray film (Kodak, XBT-1). Densitometric analysis was performed using ImageJ, and the protein expression of CD86 and CD206 was normalized to GAPDH.

### Scaffold fabrication and characterization

#### Scaffold design and assembly

The 3D-printed scaffolds were produced using a 3D bioplotter (EnvisionTEC, Germany). PCL pellets (molecular weight 80,000; Sigma-Aldrich) were air-dried overnight at 37 °C, placed into a stainless-steel cartridge equipped with a 300-μm metal tip, and heated to 140 °C for 6 h in the bioplotter’s high-temperature module. Scaffold fabrication was performed under a pneumatic pressure of 3.5 ± 0.5 bar, with a controlled deposition speed of 0.2 ± 0.1 mm/s and a platform temperature maintained at 20 °C. Successive layers of PCL were deposited in 0°, 60°, and 120° orientations, following established protocols [36]. Cylindrical scaffolds 14 mm in diameter and 5 mm in thickness were designed and generated using Bioplotter RP and VisualMachines software (EnvisionTEC). Following fabrication, all PCL scaffolds were sterilized by soaking twice in isopropanol for 30 min and then exposed to UV radiation for 30 min. The preparation of CDM@Fibrin gel was achieved by mixing fibrinogen solution (30 mg/ml), CDM (10 mg/ml), and thrombin solution (250 U/ml) in a 10:10:1 proportion. As a control, fibrin gel was produced by mixing fibrinogen solution (30 mg/ml), PBS, and thrombin solution (250 U/ml) in a 10:10:1 proportion. Hybrid scaffolds were made by quickly filling the mixed solution into the pores of PCL scaffolds using a custom-made device before being transferred to 37 °C for 30 min until polymerization was complete. Based on the previously reported porosity (approximately 50%) and volume of our 3D-printed PCL scaffolds, the nominal CDM input per scaffold was estimated to be approximately 1.83 mg based on scaffold volume, porosity, and pre-gel composition. The reported amount represents the nominal CDM input based on scaffold pore volume and precursor composition; actual retained mass/loading efficiency was not directly quantified. The homogeneity of gel filling was confirmed both visually and by subsequent SEM.

#### Scanning electron microscopy

The hybrid scaffolds were lyophilized and bisected with a surgical blade to reveal the cross-sectional area before the SEM (Zeiss Merlin) morphological assessment. Before obtaining the SEM images, the scaffolds were sputter coated with a thin layer of gold. The operating voltage was set at 5 kV.

### Animal studies

#### Rat subcutaneous implantation model

Male Sprague–Dawley rats (~200 g; supplied by Zhejiang Vital River Laboratory Animal Technology, China) were anesthetized by intraperitoneal administration of 4% pentobarbital sodium at a dose of 40 mg/kg. Each animal received 3 randomly assigned subcutaneous implants: blank, Fibrin/PCL, and CDM@Fibrin/PCL. Rats were euthanized at either 7 or 28 d following implantation. Harvested samples were fixed in 10% neutral buffered formalin at 4 °C for 24 h, then processed for paraffin embedding. Tissue sections of 5 μm thickness were prepared and subjected to H&E staining. Each group included 4 animals per time point.

#### Femoral condyle bone defect model

A rat femoral epicondylar defect model, as previously reported [37], was employed for this study. Briefly, male Sprague–Dawley rats weighing 300 to 350 g were anesthetized via intraperitoneal injection of 4% pentobarbital sodium (40 mg/kg). A sterile field was established and 1% lidocaine was administered locally. After making a skin incision, the lateral femoral epicondyle was accessed through the intermuscular space between the vastus lateralis and hamstring muscles. A bicortical tunnel was created using a 3.0-mm-diameter electric drill, ensuring continuous cold saline irrigation and suction to prevent thermal injury and remove bone debris. The periosteum overlying the tunnel was excised prior to scaffold implantation. Animals were randomly assigned to 1 of 3 groups: blank, Fibrin/PCL, and CDM@Fibrin/PCL. Meloxicam was provided intramuscularly for postoperative analgesia for 3 consecutive days. Each group included 4 animals per time point for ex vivo micro-computed tomography (micro-CT) and histological analyses, with 3 additional animals per group utilized for RNA-sequencing (RNA-seq) analysis at the 4-week time point.

#### Steroid-associated osteonecrosis model and CD

SAON was induced using a validated LPS-glucocorticoid protocol adapted from prior studies [38]: Briefly, rats received LPS (0.2 mg/kg, intravenously, day 0), followed by high-dose methylprednisolone acetate (100 mg/kg, intramuscularly, days 1 to 3) with additional daily maintenance dosing (40 mg/kg, intraperitoneally, days 4 to 28), to establish osteonecrosis. Development of SAON was confirmed in a sentinel cohort by histology/micro-CT at 2 weeks. CD was performed at week 2 post-induction: a 2.8-mm drill bit was advanced into the necrotic region to decompress the lesion. Animals were randomized to CD alone or CD + CDM@Fibrin/PCL (scaffold gently seated within the decompression defect). Wounds were closed, and standard postoperative care was provided. Endpoints for analysis were at 4 weeks post-procedure. Each group included 5 animals for ex vivo micro-CT and histological analyses, 4 for Microfil vascular perfusion scanning, and 3 for RNA-seq analysis.

#### Animal welfare and ethics

All procedures were approved by the Institutional Animal Care and Use Committee (IACUC) of The Affiliated Panyu Central Hospital, Guangzhou Medical University (PYRC-2023-058) and conformed to national regulations and Animal Research: Reporting of In Vivo Experiments (ARRIVE) guidelines. Humane endpoints, environmental enrichment, and analgesia were implemented. Euthanasia was performed under deep anesthesia followed by CO_2_ overdose per IACUC.

### Ex vivo analysis

#### Micro-CT

At 2 and 4 weeks postoperatively, rats were euthanized using carbon dioxide, and the femurs were collected for analysis. High-resolution micro-CT (SkyScan 1276; Bruker) was utilized to assess bone regeneration within the implanted hybrid scaffolds, with scans performed at an isotropic voxel size of 15 μm (85 kV and 200 μA). The region of interest (ROI) was defined to match the dimensions of the scaffold (diameter: 3.0 mm; height: 5 mm). Thresholding was standardized across samples. Quantitative assessment included measurements of bone volume to total tissue volume ratio (BV/TV), trabecular thickness (Tb.Th), and trabecular number (Tb.N). Operators were blinded to groups. For the SAON model, the same protocol was applied.

#### Histology and immunofluorescence

After micro-CT, samples were decalcified in 10% EDTA (pH 7.4, 2 to 4 wk at 4 °C, frequent changes), paraffin embedded, and sectioned (5 μm). H&E staining was performed by standard protocols. For immunofluorescence, antigen retrieval was performed in citrate buffer (pH 6.0). Sections were blocked (5% bovine serum albumin), incubated with anti-CD86 (1:100; Abcam, Ab220188) and anti-CD206 primary antibodies (1:100; Abcam, Ab64693) overnight at 4 °C, followed by appropriate fluorophore-conjugated secondary antibodies and DAPI. Images were captured with identical exposure across groups. Quantification of marker intensity (mean fluorescence intensity) and positive area fraction was done in ImageJ within a standardized ROI by 2 blinded observers, averaging ≥6 fields/section and ≥3 sections/sample.

#### Three-dimensional vascular imaging (SAON model)

At the 4-week endpoint, rats were euthanized with CO_2_ followed by a rapid laparotomy, and the inferior vena cava was transected. The descending aorta was cannulated, allowing the vasculature to be flushed first with cold 0.9% saline containing heparin sodium (100 U/ml), and then with 10% neutral buffered formalin. Subsequently, 10 ml of silicone rubber compound (Microfil MV-122; Flow Tech, Carver, MA) was injected for vascular perfusion. Perfusion was confirmed by the appearance of a yellow coloration in the intestinal mucosa and distal limbs. Specimens were stored at 4 °C overnight to allow complete polymerization of the silicone rubber in accordance with the manufacturer’s recommendations. The harvested femurs were then fixed again in 10% neutral formalin for 48 h and decalcified in 10% EDTA (Sigma, USA) for 4 weeks. High-resolution micro-CT imaging was performed to acquire images. Vascular volume within the defect ROI was quantified as vessel volume fraction (%) using CTAn (Scanco) with a standardized segmentation pipeline.

#### Immunohistochemistry for vascularization biomarker CD31

To assess the expression of CD31, immunohistochemistry was performed on tissue sections. The protocol utilized a standardized procedure including wax stripping, antigen retrieval via EDTA microwave treatment, and sequential incubation with a primary antibody targeting CD31 (1:200; Abcam, Ab182981). The staining was followed by incubation with secondary antibody (1:2,000; Abcam, Ab205718), 3,3′-diaminobenzidine (DAB) chromogen, and hematoxylin for nuclear counterstaining. The resulting stained sections were examined under a microscope, and the expression levels were qualitatively assessed based on the intensity and distribution of the brown DAB staining. For quantitative analysis, at least 3 fields were randomly selected from 2 tissue sections per sample, and CD31-positive staining was quantified using ImageJ software. For each image, the DAB-positive area percentage was calculated as follows: (DAB-positive pixels / total pixels) × 100. In addition, the microvessel count was defined as the number of discrete DAB-positive structures with an area greater than 10 pixels.

Data distribution was assessed for normality using the Shapiro–Wilk test, and homogeneity of variance was evaluated using the Levene test. Because the data were not normally distributed in either group (*P* < 0.001 for both groups) and variances were unequal (Levene test, *P* = 0.0164), between-group comparisons were performed using the nonparametric Mann–Whitney *U* test. To maintain consistency in terms of result presentation, data are presented as mean ± standard deviation (SD).

### Transcriptomic profiling

#### RNA extraction and library preparation

Bone defect tissues (and surrounding interface) were harvested into RNase-free tubes, immediately snap frozen in liquid nitrogen, and stored at −80 °C. Total RNA was extracted using TRIzol (Thermo Fisher Scientific) with on-column DNase digestion. RNA quality was assessed using a bioanalyzer (Tanon, China); samples with RNA Integrity Number ≥7 were used. Poly(A) mRNA was enriched and libraries were prepared with the ABclonal mRNA-seq Lib Prep Kit (ABclonal, China) according to the manufacturer’s protocol, indexed, and pooled.

#### RNA-seq and data analysis

Sequencing was performed on the NovaSeq 6000 (Illumina/BGI, USA) to generate paired-end 150-bp reads with a depth of 25 million reads/sample. Adapters and low-quality bases were trimmed using Trim Galore! (v0.6.7) based on Cutadapt (v3.4). Reads were aligned to the rat reference genome (mRatBN7.2) using STAR. Gene-level counts were obtained with featureCounts. Normalization and differential expression analyses were performed in DESeq2. Differentially expressed genes (DEGs) were defined as false discovery rate (Benjamini–Hochberg) <0.05 and |log_2_ fold change| ≥0.58. Data visualization included heatmaps (*z*-score of normalized counts) and volcano plots.

#### Functional enrichment analyses

GO (biological process, cellular component, and molecular function) and KEGG pathway enrichment of DEGs were conducted using clusterProfiler (R), with Benjamini–Hochberg-adjusted *P* <0.05 considered significant. Redundant terms were reduced where appropriate. Enrichment plots and pathway maps were generated in R.

### Statistical analysis

All quantitative data are presented as mean ± SD. Statistical analysis was performed using GraphPad Prism 9.0 (GraphPad Software Inc.). Comparisons among multiple groups were analyzed by one-way analysis of variance followed by Tukey post hoc test unless otherwise specified. Image-based quantifications, including immunofluorescence intensity and Western blot densitometry, were performed in ImageJ by blinded assessors where applicable. A value of *P* <0.05 was considered statistically significant. Unless otherwise stated, all in vitro experiments were performed in 3 independent biological replicates, with technical triplicates included within each experiment.

## Results

### Characterization of CDM

**Fig. 2.**
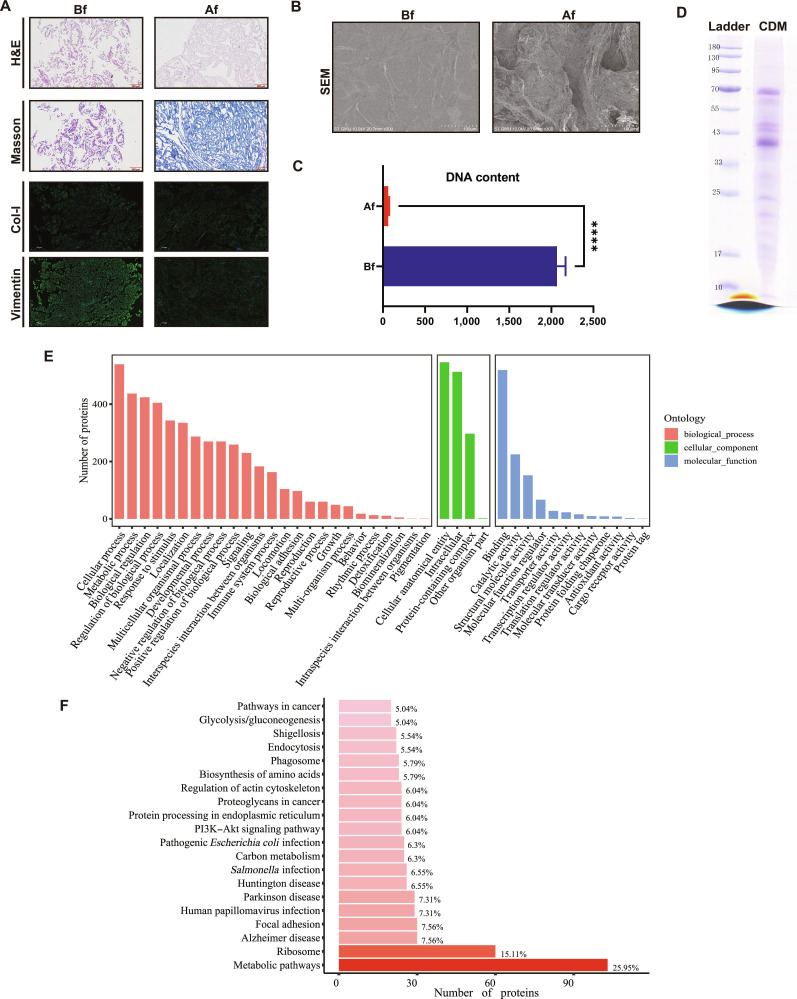
Characterization of MSC-derived CDM. (A) Hematoxylin and eosin (H&E) and Masson’s trichrome staining of MSC sheets before and after decellularization; nuclei (purple) are present in native sheets but absent in CDM, while collagen matrix (blue) remains. Immunofluorescence for collagen I (Col-I, green) and vimentin (red) in native sheets (left) versus CDM (right); DAPI (blue) highlights nuclei only in the native sample. (B) Scanning electron microscopy (SEM) images of MSC sheets before and after decellularization. (C) Quantification of residual DNA content before and after decellularization. (D) SDS–PAGE of CDM protein extract (Coomassie stain). (E) Enriched Gene Ontology (GO) biological processes (e.g., extracellular matrix organization and cell adhesion) among CDM proteins. (F) Enriched Kyoto Encyclopedia of Genes and Genomes (KEGG) pathways (e.g., metabolic pathways, focal adhesion, and PI3K–Akt signaling) were identified in the CDM proteome. Data are presented as mean ± SD. Statistical significance is indicated as ****P < 0.0001. Bf, before decellularization; Af, after decellularization.

To evaluate the efficacy of the decellularization process, we first performed H&E and Masson’s trichrome staining. The resulting images confirmed a complete absence of visible cell nuclei, suggesting that the DNA had been successfully cleared while the primary ECM architecture remained intact. This preservation was further supported by immunofluorescence staining, which showed that key structural proteins, specifically collagen and vimentin, were partially retained within the CDM. Crucially, DAPI staining revealed minimal residual nucleic acid content, while the direct DNA quantification demonstrated approximately 50 ng/mg dry weight. Such low levels indicate that the disruption and subsequent removal of genetic material were highly efficient (Fig. 2A and C).

Further structural insights were gained through SEM. The CDM exhibited an intricate, well-preserved fibrous architecture characterized by a notably rough and textured surface. This dense, interconnected mesh is typical of collagenous frameworks, featuring a homogeneous distribution of micropores. From a morphological standpoint, the total lack of discernible cellular debris reinforces the success of the decellularization protocol (Fig. 2B).

In contrast, without decellularization, SEM imaging captured a markedly different landscape. The cells displayed a robust, sprawling morphology, integrating themselves into a diffuse network of fibrillar structures. This topography appeared somewhat smoother than the matrix alone (Fig. 2B).

Finally, we utilized SDS–PAGE to profile the protein composition of the CDM (Fig. 2D). The analysis revealed a broad spectrum of protein bands across various molecular weights. This heterogeneous distribution reflects the complex protein composition retained within the matrix and is consistent with the bioactive nature of CDM.

### GO and KEGG enrichment analysis of CDM

A total of 549 proteins were identified in the CDM by LC/MS. The most abundant proteins included Fibronectin (score = 51,081, exponentially modified protein abundance index = 4.31), Myosin-9 (exponentially modified protein abundance index = 9.01), and various collagen subtypes (collagen VI and XII). Notably, growth-factor-binding proteins such as heparan sulfate proteoglycan 2 (Perlecan) and transforming growth factor-beta-induced protein were detected, which are known to modulate bone morphogenetic protein (BMP)/TGF-β signaling and promote osteogenic differentiation. These results suggest that the pro-osteogenic capacity of CDM may be attributed to its rich content of cell adhesion molecules, collagenous matrix proteins, and growth-factor-interactive components (Fig. S1). The proteomic profile of the CDM revealed a diverse array of proteins involved in biological processes, cellular components, and molecular functions critical for bone regeneration. The enrichment of proteins annotated to cellular and metabolic processes indicates that the CDM retains a biologically complex proteomic signature that may influence cell behavior during repair. Additionally, enriched proteins related to protein-containing complexes and binding proteins indicate that the CDM provides a structural framework and signaling hubs necessary for cell adhesion, cell–matrix interactions, and intracellular signaling (Fig. 2E).

Notably, proteins associated with immune system processes were also enriched, suggesting a potential role for the CDM in modulating inflammation—an essential component of the initial phase of bone healing—by regulating immune cell activity within the regenerative microenvironment.

KEGG pathway enrichment analysis identified several significantly enriched pathways within the CDM proteome, including metabolic pathways (25.95%), ribosome (15.11%), Alzheimer disease (7.56%), focal adhesion (7.56%), human papillomavirus infection (7.31%), and Parkinson disease (7.31%) (Fig. 2F). This enrichment profile provides valuable insights into potential mechanisms by which the CDM promotes bone regeneration. These findings suggest that the CDM contains diverse matrix-associated components that may participate in cell–matrix signaling and tissue remodeling. Furthermore, the presence of proteins involved in focal adhesion pathways highlights the potential role of the CDM in regulating cell adhesion, migration, and differentiation, providing essential cues to guide cellular behavior and support osteogenic differentiation.

Additionally, enrichment of the phosphatidylinositol 3-kinase (PI3K)–Akt signaling pathway underscores the potential supportive role of the CDM in osteoblast survival, proliferation, and differentiation—processes crucial for effective bone regeneration.

### Biocompatibility of CDM as supplementary substrate

**Fig. 3.**
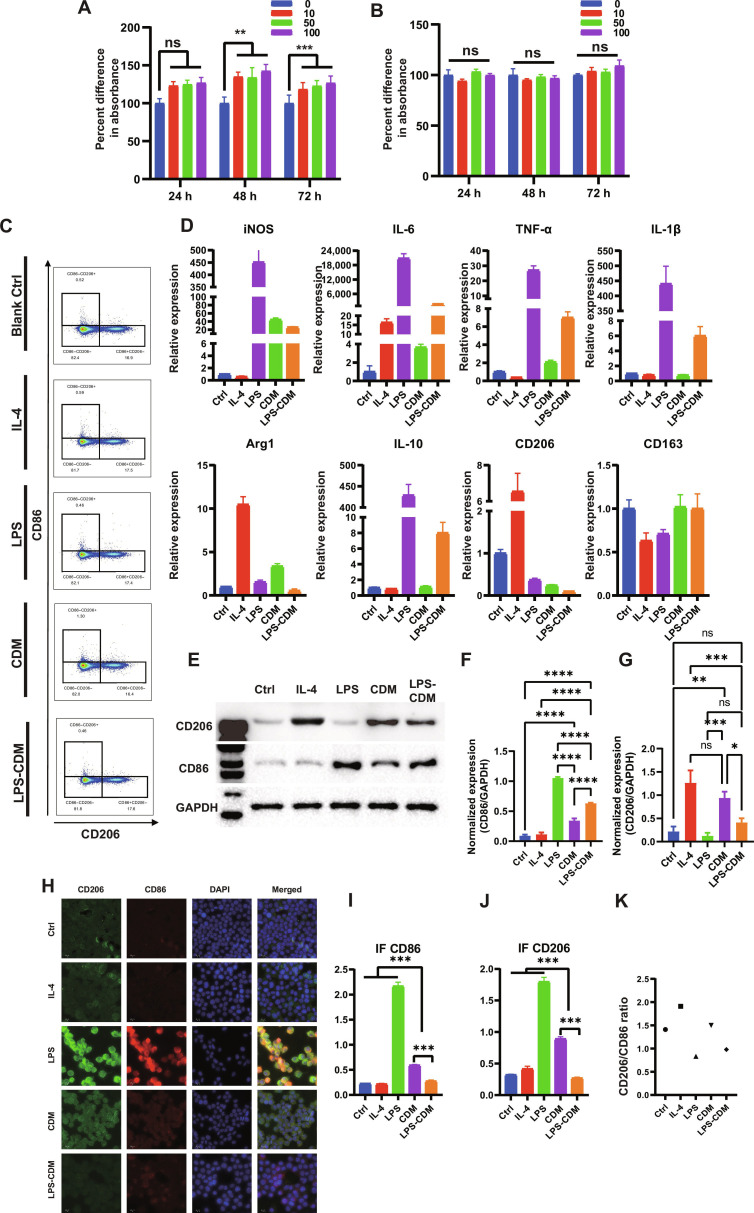
Solubilized CDM is biocompatible and modulates macrophage activation in vitro. (A) Cell Counting Kit-8 (CCK-8) assay showing relative viability/metabolic activity of UE7T-13 cells cultured with different concentrations of CDM (0, 10, 50, and 100 μg/ml) for 24, 48, and 72 h. (B) CCK-8 assay showing relative viability/metabolic activity of RAW264.7 macrophages under the same CDM concentrations and time points. (C) Representative flow cytometry plots of RAW264.7 macrophages stained for CD86 and CD206 under control, IL-4, LPS, CDM, and LPS-CDM conditions. (D) Real-time quantitative-polymerase-chain-reaction analysis of macrophage-polarization-associated genes in RAW264.7 cells under the indicated treatment conditions. (E) Representative Western blots of CD206, CD86, and GAPDH in RAW264.7 cells under the indicated treatments. (F and G) Densitometric quantification of CD86/GAPDH and CD206/GAPDH from Western blot analyses. (H) Representative immunofluorescence images of CD206 (green), CD86 (red), and nuclei (DAPI, blue) in RAW264.7 cells under the indicated conditions. (I to K) Quantitative analysis of fluorescence intensity ratios, including CD86/DAPI, CD206/DAPI, and CD206/CD86. IF, immunofluorescence. Scale bars as indicated. Data are presented as mean ± SD. Statistical significance is indicated as ns, not significant; *P < 0.05; **P < 0.01; ***P < 0.001; ****P < 0.0001.

Following our previously established protocol [34], the CDM was processed into a clear solution at a concentration of 10 mg/ml. We first evaluated the biocompatibility of solubilized CDM using a CCK-8 assay in UE7T-13 mesenchymal stromal cells and RAW264.7 macrophages. In UE7T-13 cells, CDM treatment maintained high cell viability at all tested concentrations (10, 50, and 100 μg/ml) and showed increased metabolic activity relative to the untreated control at 48 and 72 h, whereas no significant difference was observed at 24 h (Fig. 3A). In RAW264.7 cells, CDM did not induce any cytotoxicity across the same concentration range or time points, and relative viability remained comparable to the control group throughout the 72-h observation period (Fig. 3B). Based on these findings, 50 μg/ml CDM was selected for the subsequent macrophage assays.

### Solubilized CDM mitigates LPS-induced macrophage activation

In our previous work, we observed that human umbilical cord MSC microtissues—integrating both cellular components and their native matrix—triggered a more potent anti-inflammatory response than simple cell suspensions, ultimately leading to superior wound healing and bone repair [29]. These earlier findings hinted that the ECM itself might play a fundamental role in orchestrating the anti-inflammatory signaling necessary for tissue regeneration.

To examine whether solubilized CDM modulates macrophage phenotype, RAW264.7 cells were analyzed by flow cytometry after treatment under control, IL-4, LPS, CDM, and LPS-CDM conditions. As expected, IL-4 increased the CD206-positive population, whereas LPS shifted cells toward a CD86-dominant phenotype. CDM alone induced only a modest phenotypic shift compared with the untreated control. In contrast, when CDM was applied after LPS preconditioning, the macrophage profile shifted away from the strongly pro-inflammatory state, with a relative reduction in CD86 dominance and partial recovery of CD206 expression (Fig. 3C). These data suggest that CDM exerts its immunomodulatory effect most clearly under inflammatory challenge.

We next assessed macrophage-polarization-associated genes by real-time qPCR. LPS robustly up-regulated the canonical M1-associated transcripts Nos2(iNOS), Il6, Tnf, and Il1b, confirming successful induction of an inflammatory phenotype (Fig. 3D). CDM alone had a comparatively limited effect on these markers, although its expression levels remained substantially lower than those induced by LPS. Notably, in the LPS-CDM group, the expression of Nos2(iNOS), Il6, Tnf, and Il1b was reduced relative to the LPS group, indicating partial attenuation of the inflammatory response. On the M2-associated side, Arg1 was strongly induced by IL-4, as expected, whereas Il10 was highest in the LPS group and remained elevated in the LPS-CDM group, which may reflect a compensatory anti-inflammatory feedback response under inflammatory stimulation. Mrc1 (CD206) expression was highest in the IL-4 group and remained low in both the CDM-alone and LPS-CDM groups, while Cd163 showed minimal variation across conditions. Taken together, the transcriptional data indicate that CDM does not act as a strong standalone inducer of a canonical M2 program, but rather moderates the LPS-driven pro-inflammatory state, particularly under inflammatory conditions.

To further validate these findings at the protein level, Western blotting was performed for CD86 and CD206 (Fig. 3E to G). Consistent with the qPCR results, LPS markedly increased CD86 protein expression, whereas IL-4 showed the highest CD206 signal. CDM alone resulted in intermediate levels of both markers. Importantly, CDM treatment after LPS stimulation reduced CD86 expression compared with the LPS group while partially restoring CD206 expression. Quantification of normalized band intensity confirmed that the CD86 expression was highest in the LPS group and significantly decreased in the LPS-CDM group, whereas CD206 protein expression was partially restored after CDM treatment of LPS-preconditioned macrophages (Fig. 3F and G). These data support the interpretation that CDM primarily acts to mitigate inflammatory macrophage activation rather than driving a complete M2 conversion in resting macrophages.

Immunofluorescence staining further corroborated these trends (Fig. 3H to K). IL-4-treated cells displayed strong CD206 staining with low CD86 intensity, whereas LPS treatment produced the opposite pattern, with prominent CD86 expression. CDM alone showed a comparatively mild effect, with no dramatic increase in CD206 over baseline. In contrast, the LPS-CDM group exhibited reduced CD86 staining relative to the LPS group, together with partial recovery of CD206 signal. Quantitative image analysis of marker intensity normalized to DAPI supported these observations: the CD86/DAPI ratio was highest in the LPS group, while the CD206/CD86 ratio was improved in the LPS-CDM condition compared with LPS alone. Collectively, the flow cytometry, qPCR, Western blot, and immunofluorescence data indicate that solubilized CDM exerts a reproducible, context-dependent immunomodulatory effect, most prominently by mitigating LPS-induced M1-like activation and partially shifting macrophage-associated marker expression toward a more pro-healing profile.

### Fabrication of CDM@Fibrin/PCL scaffold

**Fig. 4.**
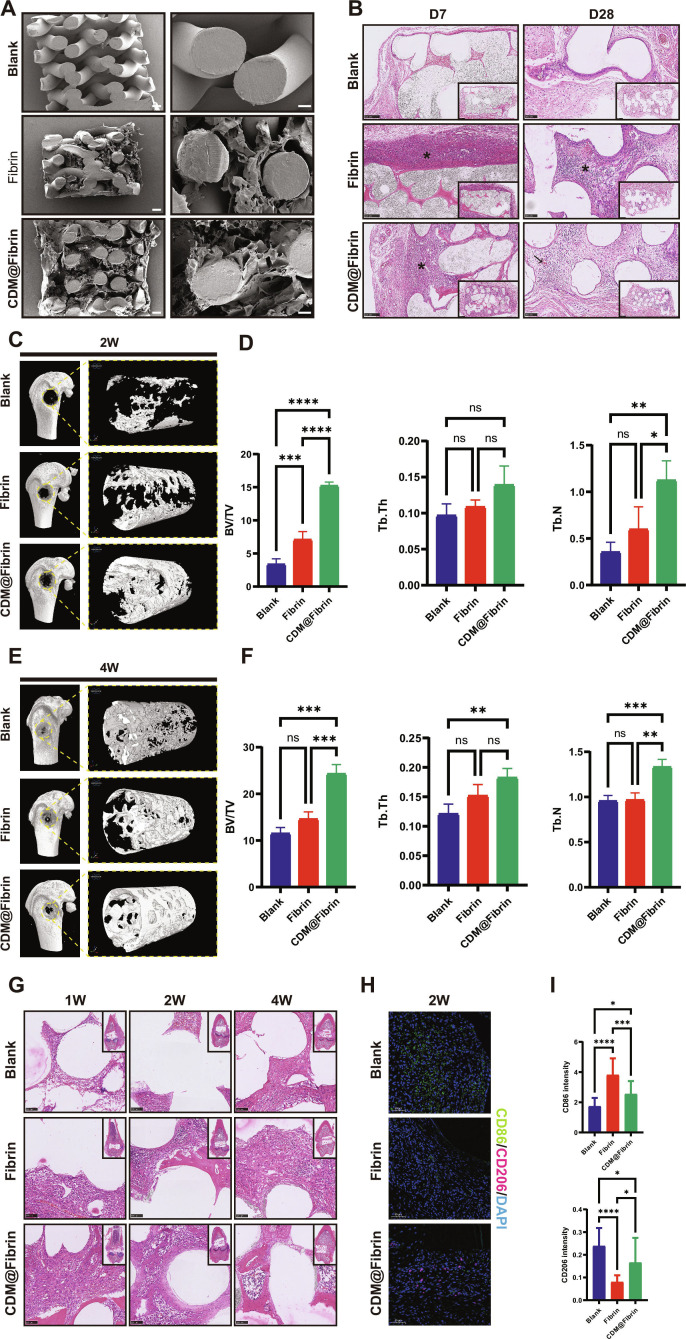
CDM@Fibrin/PCL scaffolds exhibit favorable host response and enhance bone regeneration in a rat femoral condyle defect model. (A) Representative SEM images of the 3D-printed PCL scaffolds in the blank, Fibrin/PCL, and CDM@Fibrin/PCL groups. (B) Representative H&E-staining images of explanted constructs from the subcutaneous implantation model at day 7 (D7) and day 28 (D28). Asterisks indicate infiltrated host tissue. Insets show low-magnification views of the implanted constructs. (C and E) Representative micro-computed tomography (micro-CT) reconstructions of femoral condyle defects at 2 weeks (C) and 4 weeks (E) after implantation in the blank, Fibrin/PCL, and CDM@Fibrin/PCL groups. Dashed boxes indicate the region of interest, and the right panels show enlarged 3D reconstructions of the defect area. (D and F) Quantitative micro-CT analysis at 2 weeks (D) and 4 weeks (F), including bone volume fraction (BV/TV), trabecular thickness (Tb.Th), and trabecular number (Tb.N). (G) Representative H&E-staining images of femoral condyle defect sections at 1, 2, and 4 weeks after implantation. (H) Representative immunofluorescence staining of macrophage-associated markers in the femoral condyle defect region at 2 weeks. CD86 (green) indicates pro-inflammatory macrophage-associated signal, CD206 (red) indicates pro-healing macrophage-associated signal, and nuclei were counterstained with DAPI (blue). (I) Quantitative analysis of CD86 and CD206 fluorescence intensity in the defect region. Data are presented as mean ± SD. Statistical significance is indicated as ns, not significant; *P < 0.05; **P < 0.01; ***P < 0.001; ****P < 0.0001. Scale bars as indicated. Blank, blank PCL scaffold; Fibrin, Fibrin/PCL scaffold; CDM@Fibrin, CDM@Fibrin/PCL scaffold.

Based on our established protocol [34], the CDM@Fibrin/PCL scaffold was fabricated. SEM analysis revealed distinct structural characteristics across the scaffolds. The blank PCL scaffold exhibited interconnected pores, while the introduction of fibrin resulted in a surface coated with a fibrillar network. The integration of CDM into fibrin produced a thicker, more complex porous structure, indicating enhanced coverage of the PCL strands and pore filling, thereby providing a matrix framework conducive to cellular infiltration and growth (Fig. 4A).

To evaluate biocompatibility, a rat subcutaneous model was employed. H&E staining was performed on tissue sections at day 7 (D7) and day 28 (D28) post-implantation. The blank PCL scaffold showed limited new tissue ingrowth, with mild inflammatory infiltrates observed at early stages and loosely organized neo-tissue filling at later stages. In the Fibrin/PCL scaffold, improved early neo-tissue filling was observed; however, more granulation tissue was present at the scaffold periphery. By day 28, more organized fibrous tissue filled the scaffold pores, but cell-dense granulation-like tissue and inflammatory cell infiltration persisted surrounding and within the scaffold (Fig. 4B).

In contrast, the CDM@Fibrin/PCL group demonstrated the most robust tissue integration at both early and late stages. At early stages, mild inflammatory granulation tissue was observed around the scaffold, which subsided by late stages, leaving mature tissue ingrowth within the scaffold pores (Fig. 4B).

### In vivo bone regeneration of CDM@Fibrin/PCL scaffold

Following the established rat femoral condyle bone defect model, scaffolds were implanted to evaluate bone regeneration. Blank PCL scaffolds, Fibrin/PCL scaffolds, and CDM@Fibrin/PCL scaffolds were tested.

At 2 weeks, the CDM@Fibrin/PCL group exhibited significantly higher bone formation compared to the blank and Fibrin/PCL groups. The blank scaffolds showed minimal new bone formation, while the Fibrin/PCL scaffolds demonstrated moderate regeneration (Fig. 4C and D). By 4 weeks, this trend persisted, with the CDM@Fibrin/PCL scaffolds displaying greater bone density and uniformity than the other groups. The Fibrin/PCL scaffolds maintained intermediate regeneration levels, whereas the blank scaffolds consistently showed the least bone formation (Fig. 4E and F).

Micro-CT quantification revealed that the CDM@Fibrin/PCL scaffolds had significantly higher BV/TV values than the blank and Fibrin/PCL scaffolds at both time points (Fig. 4D and F). However, differences in Tb.Th were negligible and not statistically significant (Fig. 4D and F). In contrast, the CDM@Fibrin/PCL group showed significantly higher Tb.Ns, indicating superior bone structure complexity and density (Fig. 4D and F).

Immunofluorescence staining at 2 weeks post-implantation detected CD86-positive pro-inflammatory macrophage-associated signal (green), CD206-positive pro-healing macrophage-associated signal (magenta), and DAPI (blue, nuclei). The CDM@Fibrin/PCL scaffolds contained fewer CD86-positive cells and a relatively higher CD206-associated signal than Fibrin/PCL alone (Fig. 4H). Immunofluorescence quantification revealed distinct macrophage immune-marker profiles across the scaffold groups (Fig. 4I). The Fibrin/PCL scaffold elicited a strong pro-inflammatory response, characterized by significantly elevated CD86 intensity (*P* < 0.0001 vs. blank, *P* < 0.001 vs. CDM@Fibrin/PCL) and reduced CD206 intensity (*P* < 0.0001 vs. blank, *P* < 0.05 vs. CDM@Fibrin/PCL). Conversely, the incorporation of CDM into the fibrin scaffold (CDM@Fibrin/PCL) significantly attenuated the pro-inflammatory response (*P* < 0.001 vs. Fibrin/PCL) and enhanced the pro-regenerative response (*P* < 0.05 vs. Fibrin/PCL), suggesting a shift toward a more favorable immunomodulatory microenvironment compared to Fibrin/PCL alone.

### Gene expression patterns and biological pathways in a rat bone defect model

**Fig. 5.**
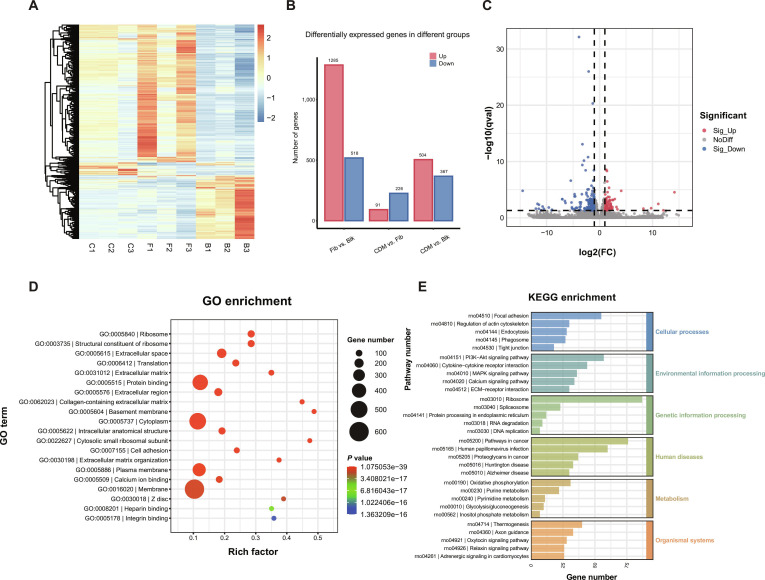
Transcriptomic profiling of the regenerative microenvironment in the rat femoral condyle defect model. (A) Heatmap showing hierarchical clustering of gene expression profiles in CDM@Fibrin/PCL (C1 to C3), Fibrin/PCL (F1 to F3), and blank PCL scaffold (B1 to B3) groups. (B) Number of differentially expressed genes (DEGs) identified among scaffold-group comparisons. (C) Volcano plot showing significantly up-regulated and down-regulated genes in the CDM@Fibrin/PCL versus Fibrin/PCL comparison. (D) GO enrichment analysis of DEGs, highlighting ECM remodeling, cell adhesion, and translation-related terms. (E) KEGG pathway enrichment analysis of DEGs, showing enrichment in pathways associated with focal adhesion, PI3K–Akt signaling, ECM–receptor interaction, cytoskeletal regulation, and metabolic activity. Blk, blank PCL scaffold; Fib, Fibrin/PCL scaffold; CDM, CDM@Fibrin/PCL scaffold.

The heatmap (Fig. 5A) illustrates distinct gene expression profiles across 3 scaffold groups: CDM@Fibrin/PCL (C1, C2, and C3), Fibrin/PCL (F1, F2, and F3), and blank PCL scaffold (B1, B2, and B3). Clear clustering indicates unique transcriptomic signatures for each scaffold type, highlighting specific biological responses elicited by the different materials.

Quantitative analysis of DEGs (Fig. 5C) showed notable differences among the groups. The comparison between Fibrin/PCL and blank scaffolds revealed the highest number of DEGs, with 1,285 genes significantly up-regulated and 518 genes down-regulated, demonstrating substantial transcriptional changes due to fibrin incorporation. In contrast, the comparison between CDM@Fibrin/PCL and Fibrin/PCL scaffolds identified fewer DEGs, with 91 genes up-regulated and 226 genes down-regulated, suggesting more nuanced biological differences introduced by the CDM component. When comparing CDM@Fibrin/PCL to the blank scaffold, an intermediate number of DEGs was observed (504 up-regulated and 367 down-regulated), indicating significant transcriptomic shifts relative to the pure scaffold.

The volcano plot (Fig. 5B) further visualizes the differential gene expression between CDM@Fibrin/PCL and Fibrin/PCL scaffolds based on statistical significance [−log10(qval)] and fold change [log2(FC)]. Several significantly up-regulated (red dots) and down-regulated (blue dots) genes were observed, confirming that CDM incorporation leads to meaningful transcriptional alterations compared to fibrin alone.

GO enrichment analysis (Fig. 5D) highlights biological pathways impacted by scaffold implantation. Highly enriched GO terms included extracellular space, ECM, and collagen-containing ECM, underscoring prominent ECM remodeling and structural organization. Additionally, ribosome and translation-related terms were significantly enriched, indicating active protein synthesis and cellular proliferation processes. Terms related to protein binding, integrin binding, and cell adhesion were also significantly represented, reflecting enhanced cellular interactions and adhesion signaling pathways.

KEGG pathway enrichment analysis (Fig. 5E) identified significantly affected pathways categorized into functional groups. In cellular processes (blue), focal adhesion, regulation of actin cytoskeleton, endocytosis, phagosome, and tight junction pathways were enriched, suggesting improved cellular adhesion, cytoskeletal dynamics, and cellular internalization processes. Environmental information processing pathways (green), including PI3K–Akt signaling, cytokine–cytokine receptor interaction, MAPK signaling, calcium signaling, and ECM–receptor interaction, underline active extracellular signaling, inflammation modulation, and cellular communication. Genetic information processing (green) pathways, such as ribosome function, protein processing in the endoplasmic reticulum, RNA transport, and RNA degradation, indicate enhanced cellular activity, transcriptional regulation, and protein synthesis. Pathways related to metabolism (brown), including oxidative phosphorylation, purine metabolism, pyrimidine metabolism, and inositol phosphate metabolism, suggest increased metabolic activity supporting tissue regeneration. Finally, organismal systems (orange) pathways, notably thermogenesis, cytokine signaling, insulin signaling, and adrenergic signaling, highlight broader physiological responses possibly linked to improved tissue repair and regeneration.

Collectively, these RNA-seq enrichment results reveal distinct transcriptomic profiles among the scaffold groups, suggesting that the CDM@Fibrin/PCL scaffold is closely associated with changes in ECM remodeling, cellular adhesion, signal transduction, metabolic activity, and systemic physiological signaling. These molecular findings align closely with previously observed histological and micro-CT outcomes, providing transcriptomic associations that are consistent with the enhanced regenerative profile observed in the CDM@Fibrin/PCL group.

### Bone regeneration of CDM@Fibrin/PCL scaffold in a SAON model

**Fig. 6.**
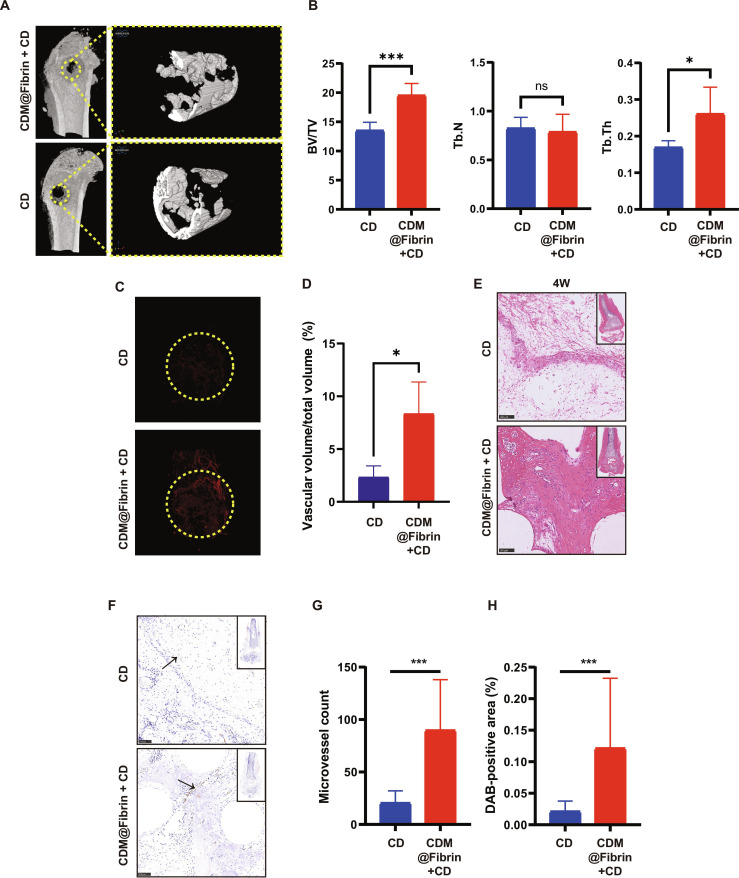
CDM@Fibrin/PCL scaffold enhances bone repair and vascularization in the SAON model. (A) Representative 3D micro-CT reconstructions of femoral condyle defects in rats treated with core decompression (CD) alone or CDM@Fibrin/PCL scaffold implantation combined with CD. Dashed circles indicate the defect region, and enlarged 3D views are shown on the right. (B) Quantitative micro-CT analysis of bone regeneration, including bone volume fraction (BV/TV), trabecular number (Tb.N), and trabecular thickness (Tb.Th). (C) Representative 3D vascular imaging of the defect region after Microfil perfusion, showing increased perfused vascular signals in the CDM@Fibrin/PCL + CD group compared with CD alone. Dashed circles indicate the analyzed region of interest. (D) Quantification of perfused vascular volume. (E) Representative H&E staining of defect sections at 4 weeks. Insets show low-magnification views of the femoral condyle region. (F) Representative endothelial-marker immunohistochemical staining in the defect region. Arrows indicate positively stained microvessel-like structures. Insets show low-magnification views. (G and H) Quantification of CD31-positive microvessel count (G) and CD31-positive staining area (H) in the defect region. Scale bars as indicated. Data are presented as mean ± SD. Statistical significance is indicated as ns, not significant; *P < 0.05; ***P < 0.001; ****P < 0.0001. CDM@Fibrin + CD, CDM@Fibrin/PCL scaffold-augmented core decompression.

3D micro-CT reconstructions demonstrate differential bone regeneration outcomes in femoral condyle defects. The CDM@Fibrin/PCL + CD treatment group exhibited superior regenerative capacity, characterized by a denser bone formation with uniform trabecular architecture and seamless integration at the defect margins. In contrast, the control (CD) group showed limited bone regeneration, with sparse trabecular networks and incomplete defect closure (Fig. 6A). Quantitative micro-CT analysis revealed significant differences in bone regeneration parameters between treatment groups (Fig. 6B). The CDM@Fibrin/PCL + CD group demonstrated a substantially greater bone volume fraction compared to the CD-only control (*P* < 0.001), demonstrated higher efficacy in promoting new bone formation. While Tb.N showed no significant intergroup difference (*P* > 0.05), the CDM@Fibrin/PCL + CD group exhibited markedly increased Tb.Th (*P* < 0.05). These results collectively indicate that the scaffold preferentially enhances bone volume and structural quality (through increased Tb.Th) rather than trabecular number, facilitating bone maturation and mechanical competence.

Analysis of vascularization and histological outcomes revealed improved regenerative potential in the CDM@Fibrin/PCL + CD group compared to the CD control. Representative 3D reconstructions (Fig. 6C) demonstrated a denser and more extensive vascular network within the defect region of the CDM@Fibrin/PCL + CD group, whereas the CD group exhibited minimal vascularization. Quantitative assessment confirmed a marked increase in vascular volume (*P* < 0.05), supporting an association with enhanced vascularization within the defect region (Fig. 6D). Histological evaluation at 4 weeks further highlighted these differences: The CD group displayed sparse, disorganized fibrous tissue with minimal bone formation, while the CDM@Fibrin/PCL + CD group exhibited well-organized bone regeneration, characterized by mature bone matrix deposition and robust cellular infiltration (Fig. 6E). To further assess vascular-associated repair, endothelial-marker CD31 immunohistochemical staining was performed in the defect area (Fig. 6F to H). The CDM@Fibrin/PCL + CD group displayed a greater number of positively stained microvessel-like structures than the CD group, and quantitative analysis demonstrated significantly increased microvessel count and positive staining area (Fig. 6G and H). Together, these findings support that the CDM-containing scaffold was associated with enhanced bone repair and improved vascularization in the SAON model.

### Gene expression patterns and biological pathways in a rat SAON model

**Fig. 7.**
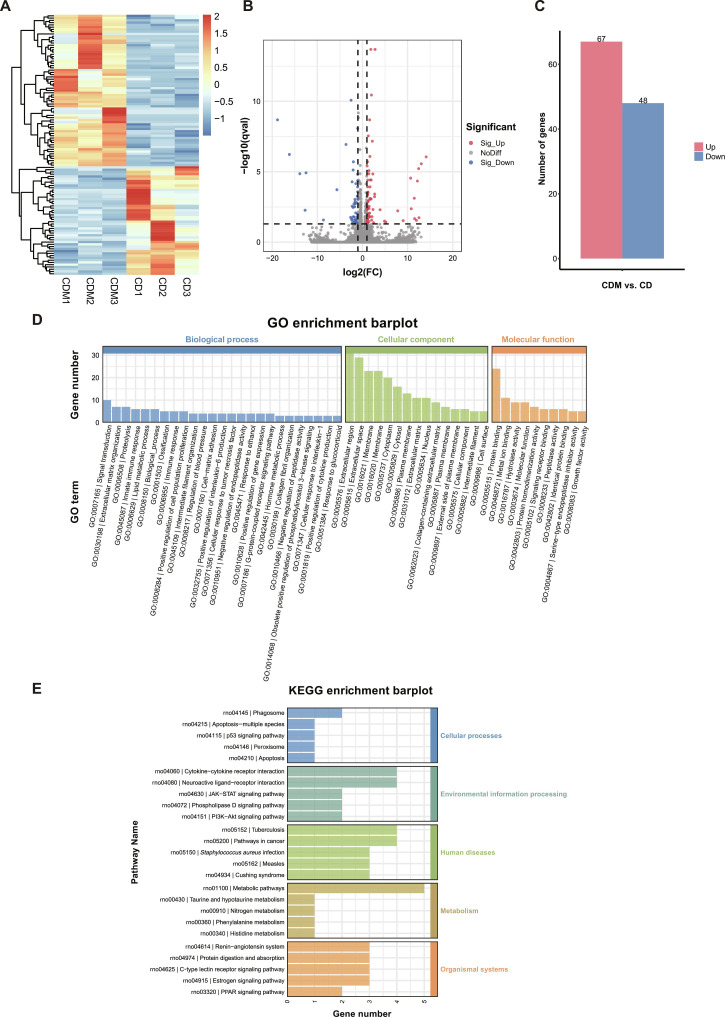
Transcriptomic changes associated with CDM@Fibrin/PCL scaffold treatment in the SAON model. (A) Heatmap showing hierarchical clustering of gene expression profiles in defects treated with CDM@Fibrin/PCL scaffold plus core decompression (CDM1 to 3) versus core decompression alone (CD1 to 3). (B) Volcano plot showing significantly up-regulated and down-regulated genes in the CDM-treated group relative to the CD group. (C) Number of DEGs identified in the CDM versus CD comparison. (D) GO enrichment analysis of DEGs, showing enrichment of terms related to immune response, inflammatory regulation, apoptosis, extracellular components, and receptor-associated functions. (E) KEGG pathway enrichment analysis of DEGs, showing enrichment in pathways related to phagosome, apoptosis, p53 signaling, cytokine–cytokine receptor interaction, Janus kinase–signal transducer and activator of transcription (JAK–STAT) signaling, PI3K–Akt signaling, and selected metabolic pathways. CD, core decompression; CDM, CDM@Fibrin/PCL scaffold-augmented core decompression; PPAR, peroxisome proliferator-activated receptor.

The heatmap visually illustrates gene expression differences between the 2 groups (CDM1, CDM2, and CDM3 vs. CD1, CD2, and CD3). Clear distinctions in gene expression patterns between the CDM and CD groups are evident, highlighting a broad transcriptomic shift associated with the CDM@Fibrin/PCL scaffold implantation compared to CD alone. A total of 115 genes were identified as DEGs between the 2 conditions, with 67 genes significantly up-regulated and 48 down-regulated in the CDM group compared to CD (Fig. 7C). Hierarchical clustering of these DEGs revealed distinct expression patterns that clearly separated the CDM samples from the CD samples (Fig. 7A). The heatmap demonstrates robust clustering, indicating consistent gene expression changes within each group. To investigate the biological significance of the DEGs, GO enrichment analysis was performed (Fig. 7D). The most enriched biological processes included “immune response”, “inflammatory response”, and “apoptotic process”. Key cellular component terms were associated with “extracellular space” and “plasma membrane”, while molecular function analysis highlighted terms such as “cytokine activity” and “receptor binding”. KEGG pathway enrichment analysis identified several significantly enriched pathways (Fig. 7E). Notably, DEGs were involved in pathways related to “phagosome”, “apoptosis”, “p53 signaling pathway”, and various immune and metabolic processes. Pathways relevant to human diseases, metabolism, and organismal systems were also represented.

## Discussion

SAON remains a formidable clinical hurdle, defined by advancing bone tissue death triggered by glucocorticoid-induced disruptions in skeletal homeostasis. These disruptions typically manifest as compromised vascular perfusion, widespread osteocyte apoptosis, and a state of chronic inflammation driven by macrophage dysregulation [39]. It is well documented that steroids cultivate a pro-inflammatory milieu where M1 macrophage polarization predominates, subsequently driving excessive osteoclast activity while stifling osteogenic potential [13,40]. At present, CD stands as the clinical benchmark for joint-preserving intervention in early-stage SAON, primarily aimed at alleviating intramedullary pressure and jump-starting revascularization. Nevertheless, the long-term success of CD is often inconsistent; nearly half of treated patients eventually face joint collapse. While adjunctive measures—including biomaterial scaffolds such as porous tantalum or 3D-printed implants—have been introduced to provide mechanical stability and improve local blood flow [41], these solutions frequently overlook the underlying immunological imbalance central to SAON. For instance, although scaffolds releasing IL-4 can encourage M2 macrophage shifts in standard bone defect models, they rarely address the specific glucocorticoid-induced M1 dominance or the severe angiogenic impairment seen in SAON [22]. Furthermore, while CDM strategies show promise for immune modulation, they often lack the structural integrity required for weight-bearing sites, leaving a gap in comprehensive preclinical validation [41]. There remains a distinct lack of research integrating CDM with CD in steroid-associated models to explore the complex interplay between inflammation, angiogenesis, and bone formation. Such gaps underscore a need for better methodological integration and translational depth in current regenerative strategies.

To address these gaps, our investigation builds upon earlier work involving MSC-laden microtissues, which previously demonstrated an ability to modulate inflammation via M2 polarization [29]. In the present study, our initial in vitro assessment showed that solubilized CDM was well tolerated by both MSCs and RAW264.7 macrophages, maintaining high cell viability and metabolic activity without evident cytotoxicity. Importantly, macrophage assays indicate that the immunomodulatory effect of CDM is context dependent. Under basal conditions, CDM alone induced only modest changes in canonical polarization markers. However, under inflammatory challenge, CDM more clearly attenuated the LPS-driven pro-inflammatory phenotype, as reflected by the partial reduction of M1-associated markers and the partial recovery of pro-healing features. This pattern was consistently supported by flow cytometry, real-time qPCR, immunofluorescence, and Western blot analyses, suggesting that CDM acts less as a strong standalone M2 inducer and more as a matrix-based regulator that tempers excessive inflammatory activation. This interpretation is consistent with the broader understanding that macrophage responses to biomaterials rarely conform to a strict binary M1/M2 framework. Rather, macrophages often occupy intermediate or transitional activation states shaped by matrix composition, inflammatory background, and tissue context. Within that framework, the marker profile observed here is most consistent with an anti-inflammatory bias rather than a complete phenotypic conversion. This distinction is important because it better reflects the in vitro data and may also be more relevant to SAON, where the therapeutic need is not necessarily to force a uniform M2 phenotype but to reduce persistent inflammatory activation sufficiently to permit vascular and osseous repair.

Then, we integrated CDM into a Fibrin/PCL framework to ensure mechanical robustness. Subsequent in vivo biocompatibility tests in a rat subcutaneous model revealed minimal cytotoxicity and excellent tissue integration. Compared to both blank and Fibrin/PCL controls, the CDM@Fibrin/PCL scaffolds showed favorable tissue integration and a less persistent inflammatory/granulation-like response. These findings suggest that CDM can beneficially influence the host response even when presented as part of a hybrid scaffold rather than as a soluble stimulus. Importantly, the in vitro and in vivo settings are mechanistically distinct: the macrophage assays evaluated the direct effect of solubilized CDM on cultured cells, whereas the implanted scaffold likely acts through a more complex combination of matrix presentation, cell infiltration, proteolytic remodeling, and local factor retention. Even so, the consistency between these datasets supports the view that CDM contributes biologically active cues that improve host–material interactions.

To further examine whether these immunomodulatory features translated into regenerative benefit, we evaluated the scaffold in a rat femoral condyle defect model. The in vivo results showed that CDM-incorporated scaffolds yielded higher volumes of new bone. Quantitative analysis, including BV/TV and trabecular numbers, were markedly elevated in the CDM@Fibrin/PCL group compared to Fibrin/PCL or PCL-only controls. Histological evaluation further confirmed that CDM-incorporated scaffolds fostered a more cellularized and mature bone matrix while dampening chronic inflammation. In parallel, immunofluorescence showed lower CD86 and higher CD206 signals in the CDM@Fibrin/PCL group compared with Fibrin/PCL alone, suggesting that CDM attenuated the pro-inflammatory tendency associated with the Fibrin/PCL scaffold and was associated with a more pro-healing local immune profile. Although these data do not establish direct causality between macrophage modulation and enhanced bone formation, they are consistent with the concept that osteoimmunomodulation contributes to the regenerative advantage of the CDM-containing scaffold.

Transcriptomic profiling in the bone defect model provided additional support for this interpretation. GO enrichment analysis indicated that the scaffolds impacted ECM remodeling, protein synthesis, and cell adhesion pathways. Furthermore, KEGG analysis highlighted enrichment in focal adhesion, cytoskeletal regulation, and PI3K–Akt signaling, suggesting that the CDM@Fibrin/PCL scaffold actively promotes cell attachment, migration, and survival [42]. At the same time, these transcriptomic data should be interpreted cautiously. Because the RNA-seq analysis was performed on dissected defect tissue, the resulting signatures reflect composite responses from multiple cell populations rather than a single dominant mechanism. Accordingly, the enrichment results are best regarded as supportive of the observed phenotype rather than definitive proof of a specific signaling axis.

The translational significance of the scaffold became more apparent in the SAON model, where the pathologic environment is both inflammatory and poorly vascularized. Compared with CD alone, CDM@Fibrin/PCL combined with CD supports vascularization-related repair and new bone formation. The increase in BV/TV, together with the gain in Tb.Th and perfused vascular volume, suggests that the scaffold supports not only greater tissue infill but also improved structural maturation within a hypovascular and inflammatory environment. This is particularly relevant for SAON because successful joint preservation depends not only on filling the decompression defect but also on re-establishing a biological milieu capable of sustaining angiogenesis and new bone formation [43]. In this regard, the present findings suggest that CDM@Fibrin/PCL serves as more than a space-filling implant; it appears to function as a microenvironment-instructive adjunct to decompression. The RNA-seq data from the SAON model further support this interpretation. DEGs between the CD and CDM-treated groups were enriched in pathways related to immune resolution and ECM organization [8,19,39]. Notably, the enrichment of p53 and phagosome signaling suggests that the scaffold may facilitate the clearance of necrotic debris while protecting osteoprogenitor cells from apoptosis [19,44]. These changes are consistent with the possibility that the scaffold alters the local inflammatory and reparative landscape after implantation. However, these findings remain associative. The current study does not identify which cell populations are primarily responsible for these transcriptomic shifts, nor does it establish whether specific pathways are directly required for the observed regenerative benefit. Future studies using cell-specific analysis and functional perturbation will be necessary to define these mechanisms more precisely.

From a biomaterials perspective, the present results support the use of MSC-derived CDM as a bioactive matrix component rather than a simple structural additive. CDM serves as a versatile natural biomaterial, typically harvested from sources like fibroblasts, endothelial cells, or MSCs [26,27,34,45,46]. Following decellularization, it can be utilized as an intact sheet or processed into hydrogel precursors for bioink formulations [47]. While all CDM variants generally support basic cell functions like adhesion and proliferation, they also retain tissue-specific biochemical signatures that mimic native microenvironments [46]. It is increasingly recognized that MSCs exert their therapeutic effects largely through paracrine-mediated immunomodulation [48]. In our scaffold, CDM was physically integrated within the fibrin phase and likely acted through a combination of direct cell–matrix interactions, local presentation of retained bioactive molecules, and gradual enzymatic remodeling after implantation. Proteomic analysis identified proteins associated with extracellular matrix organization, focal adhesion, and PI3K–Akt-related signaling, all of which are relevant to cell survival, adhesion, migration, and repair. Nevertheless, considering the in vitro data, it is more appropriate to interpret CDM as a context-responsive immunomodulatory matrix that dampens inflammatory activation and biases the local environment toward repair, rather than as a universal direct inducer of canonical M2 macrophage conversion.

The choice of PCL as the structural backbone also remains important for translational feasibility. PCL remains a staple in bone engineering due to its predictable degradation and synthetic reliability [35]. Building on our prior findings [35], similar 3D-printed PCL scaffolds featuring a 0°/60°/120° laydown architecture possess a compressive modulus suitable for cancellous bone defects. Besides, 3D printing allows us to fabricate PCL scaffolds with precise porosity, which, when hybridized with hydrogels, maintain high structural integrity [34]. Previous literature has shown that modified PCL can enhance biocompatibility and bone formation [49]. In our design, the 3D-printed PCL framework provided the load-bearing skeleton, while fibrin and CDM supplied a more permissive biological microenvironment. This division of function is advantageous in SAON, where both structural support and biological rescue are required. Our data demonstrate that the CDM@Fibrin/PCL hybrid not only excels in standard bone defects but also performs favorably in the challenging environment of a SAON model. The present data therefore support a hybrid design strategy in which a mechanically competent synthetic scaffold is combined with a matrix-derived bioactive phase to address the dual demands of orthopedic regeneration.

From a clinical perspective, these findings carry marked weight. Our cell-free CDM approach offers several distinct translational advantages over conventional therapeutic strategies. While traditional cell-based interventions, such as MSC transplantation, have demonstrated clinical promise, their broader application is constrained by poor in vivo survival, high manufacturing expenses, and rigorous regulatory scrutiny regarding safety and immunogenicity. Conversely, while single-cytokine delivery systems, such as scaffolds releasing IL-4 or BMP-2, can trigger specific cellular pathways, they are frequently limited by burst release kinetics, rapid degradation, and an inability to emulate the synergistic, multifaceted signaling essential for hierarchical tissue repair. Our solubilized CDM, by contrast, establishes a natural, biomimetic microenvironment. By preserving a reservoir of structural ECM proteins and endogenous signaling molecules, the CDM modulates the local immune landscape while concurrently supporting osteogenic and vascular reparative processes, thereby potentially reducing some of the translational and regulatory challenges associated with live-cell therapies. The “immuno-instructive” nature of CDM is particularly relevant for conditions defined by chronic inflammation, such as SAON, diabetic non-unions, or geriatric fractures. While additional work will be required to standardize manufacturing and define quality-control metrics for CDM preparations, the present findings support further exploration of CDM-integrated scaffolds as adjuncts to joint-preserving procedures.

Several limitations should be acknowledged. First, the in vivo efficacy studies were performed over a relatively short 4-week period in rodent models, which do not fully recapitulate the biomechanical demands, lesion size, and chronic disease heterogeneity seen in human SAON. Longer-term studies in larger animal models will be necessary to assess remodeling durability, scaffold degradation, and safety. Second, the in vitro macrophage experiments were conducted in RAW264.7 cells, which are useful for mechanistic screening but do not fully capture the complexity of primary macrophage responses in vivo. Third, the SAON efficacy study used a limited sample size, and therefore the observed regenerative and transcriptomic trends should be interpreted with appropriate caution. Finally, although RNA-seq provided a useful overview of pathway-level changes, these data remain descriptive and associative. We did not perform targeted validation of selected differentially expressed genes, macrophage depletion studies, or pathway inhibition experiments; accordingly, definitive causal mechanisms linking CDM to immune modulation, angiogenesis, and osteogenesis remain to be established.

In summary, this study demonstrates that incorporation of CDM into a Fibrin/PCL scaffold generates a bioactive hybrid construct with mechanical support that improves host response and promotes vascularized bone regeneration. Rather than acting as a strong standalone inducer of canonical M2 polarization, CDM appears to function as a context-dependent immunomodulatory matrix that attenuates excessive inflammatory activation and supports a more pro-healing microenvironment, particularly under inflammatory conditions. When combined with CD in a preclinical steroid-associated osteonecrosis model, the CDM@Fibrin/PCL scaffold enhanced bone formation and vascularization compared with decompression alone. These findings support the potential of CDM-based immuno-instructive scaffolds as cell-free adjuncts for joint-preserving treatment of SAON, while also highlighting the need for further mechanistic studies and long-term large-animal validation before clinical translation.

## Ethical Approval

All procedures were approved by the IACUC of The Affiliated Panyu Central Hospital, Guangzhou Medical University (PYRC-2023-058) and conformed to national regulations and ARRIVE guidelines.

## Data Availability

Data and materials would be disclosed on reasonable request.
