# Supplementary material for: A Hybrid Mesenchymal-Stem-Cell-Derived Decellularized Matrix Scaffold Supports Bone Repair and Vascular Perfusion in Steroid-Associated Osteonecrosis
Source: Biomater Res. 2026 Jun 29;30:0383. doi: 10.34133/bmr.0383 (PMC13311255; doi:10.34133/bmr.0383)
Supplement: Supplementary 1 — Fig. S1 Table S1 [file bmr.0383.f1.zip › supplemental figure with a captionlegend.docx]

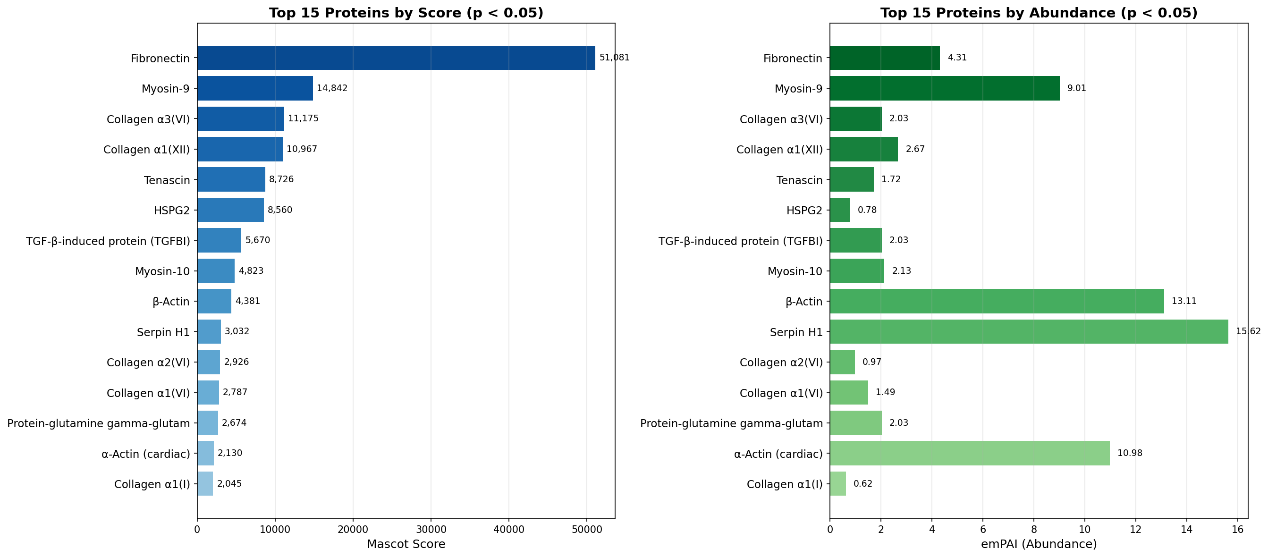


Supplementary Figure 1. Proteomic profiling of key proteins retained in the MSC-derived cell-derived matrix (CDM). Bar charts showing the top 15 significantly identified proteins in the CDM proteome ranked by Mascot score (left) and relative abundance estimated by emPAI (exponentially modified Protein Abundance Index; right) (p < 0.05).

| Gene Primer | Sequence | Product Length |
| --- | --- | --- |
| iNOS-F1 | CTGCTTTGTGCGAAGTGTCA | 150bp |
| iNOS-R1 | CCTCCTTTGAGCCCTTTGTG |  |
| IL-6-F1 | GTTGCCTTCTTGGGACTGAT | 160bp |
| IL-6-R1 | TTTCCACGATTTCCCAGAGA |  |
| TNF-α-F1 | TCTACTCCCAGGTTCTCTTCA | 90bp |
| TNF-α-R1 | CCTGGTATGAGATAGCAAATCG |  |
| Arg1-F1 | CTGACATCAACACTCCCCTG | 133bp |
| Arg1-R1 | GCAGATATGCAGGGAGTCAC |  |
| CD206-F1 | TCCCTGCCTGTTTCTCCAACCA | 304bp |
| CD206-R1 | TAAGCTTCGGCTCGTCAGCA |  |
| CD163-F1 | TGCCAAACCGTGGAGTCACA | 195bp |
| CD163-R1 | CGCTGAATCTGTCGTCGCTT |  |
| β-actin-F1 | GCTTCTAGGCGGACTGTTAC | 100bp |
| β-actin-R1 | CCATGCCAATGTTGTCTCTT |  |

95°C for 5 min; 95°C for 15 s, 60°C for 32 s (plate read), 40 cycles; Melt curve analysis: 60°C to 95°C.

Supplementary Table 1. Primer sequences of RT-qPCR.
