# Supplementary material for: A Hybrid Mesenchymal-Stem-Cell-Derived Decellularized Matrix Scaffold Supports Bone Repair and Vascular Perfusion in Steroid-Associated Osteonecrosis
Source: Biomater Res. 2026 Jun 29;30:0383. doi: 10.34133/bmr.0383 (PMC13311255; doi:10.34133/bmr.0383)
Supplement: Supplementary 1 — Fig. S1 Table S1 [file bmr.0383.f1.zip › Table 1 RT-qPCR Primer Sequence.docx]

| Gene Primer | Sequence | Product Length |
| --- | --- | --- |
| iNOS-F1 | CTGCTTTGTGCGAAGTGTCA | 150bp |
| iNOS-R1 | CCTCCTTTGAGCCCTTTGTG |  |
| IL-6-F1 | GTTGCCTTCTTGGGACTGAT | 160bp |
| IL-6-R1 | TTTCCACGATTTCCCAGAGA |  |
| TNF-α-F1 | TCTACTCCCAGGTTCTCTTCA | 90bp |
| TNF-α-R1 | CCTGGTATGAGATAGCAAATCG |  |
| Arg1-F1 | CTGACATCAACACTCCCCTG | 133bp |
| Arg1-R1 | GCAGATATGCAGGGAGTCAC |  |
| CD206-F1 | TCCCTGCCTGTTTCTCCAACCA | 304bp |
| CD206-R1 | TAAGCTTCGGCTCGTCAGCA |  |
| CD163-F1 | TGCCAAACCGTGGAGTCACA | 195bp |
| CD163-R1 | CGCTGAATCTGTCGTCGCTT |  |
| β-actin-F1 | GCTTCTAGGCGGACTGTTAC | 100bp |
| β-actin-R1 | CCATGCCAATGTTGTCTCTT |  |

95°C for 5 min; 95°C for 15 s, 60°C for 32 s (plate read), 40 cycles; Melt curve analysis: 60°C to 95°C.
